# Administrative pressure, interpersonal relationships, and teachers’ professional identity

**DOI:** 10.3389/fpsyg.2025.1505258

**Published:** 2025-06-16

**Authors:** Bao Zhu, Shiting Zhai

**Affiliations:** ^1^School of Finance and Accounting, Henan University of Animal Husbandry and Economy, Zhengzhou, China; ^2^School of Humanities and Law, Henan University of Animal Husbandry and Economy, Zhengzhou, China

**Keywords:** professional identity, administrative pressure, interpersonal relationships, secondary school teachers, order probit model

## Abstract

Understanding teachers’ professional identity is significant for promoting their professional development, cultivating a high-quality teaching force, and achieving a high-quality education system. This paper empirically analyzes the impact of administrative pressure on teachers’ understanding of professional identity based on data from the China Education Panel Survey using the order probit model to reveal the mechanisms at play in this relationship. Administrative pressure was found to inhibit teachers’ professional identity significantly. The conclusions hold after using conditional mixed regression to deal with endogeneity and robustness tests using various strategies. Heterogeneity analyses revealed that the inhibitory effect of administrative pressure was more pronounced for female teachers and teachers in rural areas. Mechanistic tests indicated two channels for this inhibition—by decreasing teachers’ job satisfaction and increasing teachers’ burnout. The moderating effects tests also showed that the inhibitory effect was relatively weaker when teachers’ interpersonal relationships were better. This study enriches and deepens our understanding of administration and teachers’ professional development, provides new empirical evidence for understanding how administrative pressure affects teachers’ professional identity, and provides a theoretical basis for educational administrations to formulate more effective educational policies and management strategies.

## 1 Introduction

Enhancing teachers’ professional identity is critical for cultivating a high-quality teaching force. This process serves as a fundamental driver to achieve high-quality development in education. The State Council of China has issued policy documents to clarify the important position of teachers in the development of education. It emphasizes that teachers are the first resource for educational development, and that the objectives and tasks of upgrading the remuneration of teachers and enhancing the attractiveness of the teaching profession (Comprehensively Deepening the Reform of Teaching Staff Construction in the New Era, 2018). In April 2022, China’ s Ministry of Education, in collaboration with seven other authorities, proposed a number of specific measures to promote the professional development of primary and secondary school teachers (New Era Basic Education Teacher Strengthening Plan, 2022). Several specific measures and implementation guarantees were proposed to promote the professional development of primary and secondary school teachers. Professional identity is a core component of teachers’ professional development and an intrinsic motivation for teachers’ self-growth (Yang, 2014), which also has a significant influence on teachers’ professional behavior tendency (Li and Yan, 2018). Teachers’ professional identity is a composite of positive perceptions, experiences, and behavioral dispositions toward their profession (Wei, 2008), in addition to being the process by which individual teachers internalize their professional roles as part of their selves; furthermore, professional identity is a psychological foundation that must be laid during professional development (Zhang et al., 2012). The study of teachers’ professional identity has important theoretical and practical significance in promoting their professional development, cultivating a high-quality teaching force, and improving the quality of both education and teaching.

There are numerous discourses in the study of teachers’ professional identity. Some studies focus on the factors influencing teachers’ professional identity. For example, Barahona and Ibaceta-Quijanes (2020) surveyed 716 Chilean teachers of English and found that administrative constraints, working conditions, and salary levels could significantly influence their professional identity. Composition of the membership of teacher appraisal organizations can directly affect teachers’ professional identity, while the substantive rewards and punishments after the appraisal can indirectly affect it (Yi and Chen, 2019). The support and attitudes of colleagues, classroom organizations, school facilities, and school leadership are also major influencing factors (Fatma and Golge, 2023).

Shaping teachers’ professional identity is equally a key concern for researchers. For example, Zhang (2018) discussed school strategies to enhance teachers’ professional identity and emphasized that stimulating teachers’ sense of value in teaching should be good at catching flash points. Cultivating a sense of professional honor by respecting teachers, optimizing the system, and cultivating both virtue and skill is another important strategy (Liang and Gao, 2020). Pre-service teacher professional training and educational practice also play an important role in teachers’ career choices and in shaping their professional identity (Wang, 2017; Jin and Li, 2019; Wang et al., 2023). Thus, although academics have explored the factors influencing teachers’ professional identity and interventions at different levels, the extent to which administrative pressure affects teachers’ professional identity and the mechanism by which it works has not yet been adequately researched.

Administrative pressure on teachers mainly stems from non-instructional matters caused by a range of administrative measures exercised by the school authorities. According to a survey conducted by People’s Education on education topics for the 2024 session, the option of “reducing the non-teaching burden on teachers” topped the list of topics for teachers with 29.32% of the votes. The administrative pressure on teachers has been increasing in recent years because of social change and education reform. Primary and secondary school teachers leaving their jobs on their own initiative or even becoming depressed due to excessive administrative pressure has occasionally occurred, and the resulting social opinion has had a negative impact on education and teaching.

In December 2019, China issued a guideline to reduce the burden on teachers at primary and secondary schools and to create a good environment for education. The guideline sought to coordinate and standardize matters of supervision, inspection, evaluation, and assessment, so teachers can concentrate on teaching and educating. However, in practice, the exercise of the rights of grass-roots education administrations continues to have a profound impact on teachers’ living conditions, which has led to increased pressure on teachers and a diminished sense of well-being and professional identity (Lu, 2020). Moreover, some empirical analyses have shown that administrative burdens have a significant effect on teachers’ physical and mental health and enthusiasm for teaching (Jiang, 2023). Accordingly, the following urgent questions are addressed in the present study. What, then, is the impact of administrative pressure on teachers’ professional identity? Is there a difference in this impact by gender and by region? How does administrative pressure affect teachers’ professional identity? What are the mechanisms and pathways?

Interpersonal relationships encompass the psychological connections and social bonds formed through individual interactions. In psychological terms, these relationships are characterized as direct psychological ties established via interactions. From a sociological perspective, interpersonal relationships denote social connections developed during various productive or living activities, including friendships, marital relationships, parent–child dynamics, classmate interactions, and teacher-student associations. These relationships serve as a primary source of emotional support and significantly influence an individual’ s mental and physical well-being (Bowling et al., 2005). In educational research, the quality of interpersonal relationships within the educational environment is recognized as a critical factor influencing students’ learning outcomes and psychological well-being. Effective communication between teachers and students significantly impacts teaching efficacy and student motivation, with a notable positive correlation between supportive teacher behaviors and student trust, which not only enhances student motivation but also fosters academic achievement (Frymier and Houser, 2000). Ruohotie-Lyhty and Moate (2016), in their study of forms of agency in the development of pre-service teachers’ professional identity, found that good interpersonal relationships promote the positive construction of professional identity. Consequently, this paper further examines the moderating role of interpersonal relationships in this context, exploring the mechanism through which administrative pressure affects teachers’ professional identity.

The marginal contributions of this paper include, first, from a theoretical perspective, a systematic study of the impact of administrative pressure on teachers’ professional identity and its mechanism of action, which enriches and deepens the relevant theories of administration and teachers’ professional development. Second, in terms of identification strategies, this paper enhances the credibility of the core finding that administrative pressure significantly inhibits teachers’ professional identity based on the use of the instrumental variables approach and adequate robustness tests. Mechanistic analysis using mediated effects modeling helped to clarify the mechanism by which administrative pressure affects teachers’ professional identity. Third, the moderating effect of interpersonal relationships was examined and tested within the framework of the study of administrative pressure on teachers’ professional identity, which can help to optimize management strategies and build a high-quality teaching force in the new era.

## 2 Theoretical analysis and research hypotheses

### 2.1 Effect of administrative pressure on teachers’ professional identity

Teachers’ professional identity is intricately linked to teacher agency, and the qualitative research paradigm continues to hold significance in this area of study. However, there has been a notable shift from qualitative to quantitative research paradigms, which necessitates the development of more robust quantitative evaluation methods (Yan, 2024). Using a quantitative approach, Li and Khairani (2025) analyzed a sample of 4,999 Chinese pre-service teachers through structural equation modeling. They found that social support and pedagogical beliefs enhance teachers’ professional identities, while culture strengthens the association between the two. Similarly, Wang et al. (2024) constructed a structural equation model based on a cross-sectional survey of 1,001 Chinese special education teachers. Their findings indicated that teachers’ professional identity positively influenced developmental agency, with work engagement partially mediating this relationship, and social support serving as a moderator. Pulsawad et al. (2025) conducted a questionnaire survey of teacher educators specializing in math and science, revealing that they were more likely to identify as general and subject matter teachers rather than as STEM teachers. This suggests a lack of sufficient support for STEM identity development within current teacher education programs. Furthermore, Pi et al. (2024) conducted a quantitative study involving 2,668 special education teachers, demonstrating that professional identity partially mediated professional development through self-efficacy, with perceived social support moderating this indirect relationship. In the field of professional identity research, quantitative methods have emerged as the dominant paradigm, driven by their scientific rigor, which is rooted in standardized measurement tools, statistical analysis, and large-sample validation. Meanwhile, quantitative research does not supplant qualitative inquiry; rather, it serves as a valuable complement. Together, these methods advance the field toward more systematic and explanatory frameworks that inform both theoretical development and practical intervention.

Teachers’ professional identity is a composite of positive perceptions, experiences, and behavioral dispositions towards their profession and professional roles; it is generated by individual teachers in relation to their profession. It affects teaching behavior and quality, as well as teachers’ level of commitment to education. A strong sense of professional identity is conducive to improving motivation and teaching quality. Teachers’ professional identity is a multifaceted and dynamic process influenced by the interaction of various factors. Existing literature indicates that research on teachers’ professional identity encompasses multiple dimensions, including age, gender, working experience, educational background, salary and marital status. Bukor (2014) highlighted the influence of personal and professional experiences on the development of teacher identity. Doğan and Erdiller Yatmaz (2016) collected data from 1,021 Turkish early childhood education teachers through simple random sampling and found that teachers’ professional identity was affected by such factors as gender, monthly income, educational level and background, job position, current work experience and marital status of teachers. Lamote and Engels (2010) examined student teachers’ professional identity by analyzing questionnaires completed by students in two teacher education colleges and found that practical classroom experience influenced students’ task orientation and that gender differences were evident in professional identity. Based on data from 608 teachers in central and western China, Qin and Liu (2023) found that female teachers had higher levels of professional identity and perceived professional learning than their male counterparts, and that principal support had a significant positive effect on teachers’ professional learning, and that teachers’ professional identity played a partly mediating role in this.

Administrative pressure on teachers primarily stems mainly from non-instructional matters caused by administrative measures exercised by the school authorities. Such pressures may come from the demands created by changes in educational policy, teaching, and learning assessment, or from the influence of the school’ s administrative system and the management style of its leaders. The impact of administrative pressure on teachers’ professional identity is reflected in several areas. First, it may have a negative impact on motivation and incentives. When faced with administrative indicators such as appraisals and rankings, teachers may feel drained and lose enthusiasm for their work, thus reducing their sense of professional identity. Second, pressure may lead teachers to choose safer and more administratively compliant approaches to teaching rather than spending more time and energy on pedagogical reform and innovation, thus further diminishing their professional identity. Finally, excessive administrative pressure may crowd out instructional work time, so teachers are forced to work on non-instructional matters and unable to concentrate on developing students. This can cause teachers to lose their belief in and enthusiasm for the profession, reduce their professional identity. In summary, the following hypothesis is proposed.

*H1*: Administrative pressure has a dampening effect on teachers’ professional identity.

### 2.2 Mechanisms of action for the effect on teachers’ professional identity

Teachers’ job satisfaction can have a significant impact on their professional identity. Job satisfaction is a general, emotionally charged feeling and perception of their job and profession, as well as their working conditions and situation (Landy, 1989). Research has shown that teachers’ job satisfaction is closely linked to professional identity (Goulet and Singh, 2002; Luo et al., 2014; Sun et al., 2022). Teachers with high job satisfaction usually love their jobs more, have a stronger sense of responsibility and mission for education, and are more willing to invest more time and energy in education. They also actively participate in various training and learning activities to continuously improve their professional competence and lay a solid foundation for their career development. When teachers receive positive feedback and have higher satisfaction in their work, it significantly increases their confidence in practicing their profession, as well as improving their sense of belonging, which in turn enhances their professional identity. However, excessive administrative pressure may increase teachers’ workload, limit their pedagogical autonomy and creativity, and lead to stress and anxiety, thus reducing job satisfaction. It is possible, then, that administrative pressure may inhibit teachers’ professional identity by reducing job satisfaction.

Burnout can also have a significant impact on teachers’ professional identity. Burnout primarily manifests in the loss of enthusiasm for work, or even an aversion to the work they are engaged in, a negative and indifferent attitude toward the work object, a lack of accurate self-efficacy judgment, and the absence of a sense of personal accomplishment (Maslach et al., 1997). Burnout is recognized worldwide as a problem that seriously affects the professional health of teachers and as a clear impediment to building a high-quality teaching force (García-Arroyo et al., 2019; Gao et al., 2023). Research has shown that teacher burnout is closely related to professional identity (Huang et al., 2021; Zhang et al., 2024). When teachers experience burnout, it may cause them to begin to doubt the value of their work and question their ability to do their job, thus reducing their professional identity. Burnout can also lead to a lack of commitment to teaching and a lack of willingness to put in extra effort to improve methods to enhance quality, which can further weaken professional identity.

Administrative pressure may increase burnout. First, it interferes with teaching work, resulting in teachers not being able to work independently in teaching, thus gradually increasing burnout. Second, administrative pressure increases workload and work intensity, as teachers must fulfill the required administrative tasks along with their daily teaching duties and student management. With long hours of intense work, burnout can grow. Finally, administrative pressure affects personal professional development. Teachers need to learn and improve their teaching continuously, and excessive administrative tasks can crowd out teachers’ time for self-improvement, which may further reduce teachers’ motivation and increase burnout. In summary, this paper proposes the following hypothesis.

*H2*: Administrative pressure can inhibit teachers’ professional identity by decreasing their job satisfaction as well as increasing their burnout.

Interpersonal relationships are an important part of how people interact and relate to each other in society. Collaboration and communication between teachers and colleagues are integral parts of their work. When teachers build good interpersonal relationships with each other, it can promote win-win cooperation and improve the effectiveness of the teacher community. Good interpersonal relationships are beneficial, because teachers to receive more emotional support and job assistance, which can reduce the perception of administrative pressure. Engaging with colleagues provides teachers with a chance to understand their colleagues and their concerns when facing administrative pressure. This helps teachers to focus more on their work of teaching, improves their work efficiency, and reduces the negative impact of administrative pressure on teachers’ professional identity. Accordingly, this paper proposes the final hypothesis.

*H3*: When teachers have better interpersonal relationships, it weakens the inhibitory effect of administrative pressure on teachers’ professional identity.

## 3 Methods

### 3.1 Sample and data

The data used in this paper were primarily from the 2014–2015 CEPS. The database is a large, nationally representative tracking program that contains information from a variety of questionnaires from teachers, students, and families. The CEPS is a comprehensive tracking survey project designed and implemented by the National Survey Research Center at Renmin University of China (NSRC). Data were reviewed by the Ethics Committee of Renmin University of China. The survey sample comprised 112 schools, 438 classes, and approximately 20,000 students within China, with respondents including students, parents, teachers, and school leaders. This paper primarily utilizes questionnaires for classroom teachers and school leaders. The classroom teacher questionnaire encompasses three main categories: basic personal information, working conditions, and educational philosophy. Basic personal information includes teachers’ age, gender, highest education level, and years of teaching experience. Working conditions cover weekly working hours, courses taught, and teaching methods employed. The educational philosophy category explores teachers’ views on educational goals, student training methods, and the importance of personalized student development in the teaching process. The school leader questionnaire also includes three categories: basic school information, teaching facilities, and education management. Basic school information comprises the school’s geographic location, size, and operational nature. Teaching facilities cover the equipment and utilization of teaching buildings, libraries, and sports facilities. The education management category includes curriculum arrangement, teaching evaluation systems, teacher development mechanisms, and related aspects. These characteristics serve as foundational variables for studying teachers’ professional identity.

### 3.2 Variable setting and description

#### 3.2.1 Dependent variable

The dependent variable was teachers’ professional identity (*identity*), which was measured by the question: “If you had to choose again, would you choose teaching as a career?” Interviewed teachers chose from three options: “1. No,” “2. Not necessarily,” and “3. Definitely.” The higher the value, the stronger the teachers’ professional identity, and the more inclined they are to remain in the teaching profession.

#### 3.2.2 Independent variable

The independent variable was administrative pressure (*adpress*), which was measured by the question: “Are you stressed by all the administrative measures of the school?” Responses were rated on a 5-point scale, where 1 means that the administrative measure is not stressful and 5 means that it is very stressful. The higher the value, the greater the administrative pressure felt by the interviewed teachers.

#### 3.2.3 Control variables

To reduce the estimation bias caused by omitted variables, we refer to Fatma and Golge’s (2023) approach, control variables related to professional identity at the level of individual and job characteristics were included. Individual characteristics included age (*age*), time teaching at the school (*tschool*), length of teaching experience (*lteaching*), marital status (*married*), how long on teaching staff (*organization*), highest level of education (*education*), attitudes toward school pay (*salary*), and awards received (*award*). Job characteristics included work hours (*hours*), teaching tasks (*tasks*), and classroom achievements (achievement). School factors may include environmental variables that also affect professional identity, so control variables related to the school environment were also included, such as the size of the school (*scale*), whether the school has a playground (*playground*), whether the school has a canteen (*canteen*), and the learning climate of the school (*climate*).

#### 3.2.4 Mediating variables

The mediating variables were job satisfaction (*satisfaction*) and burnout (*burnout*). Job satisfaction was measured using the question: “Overall, are you satisfied with your current job as a teacher” Responses were rated on a 5-point scale, where 1 means that the interviewed teacher is very dissatisfied with his/her job as a teacher and 5 means that the interviewed teacher is very satisfied. The higher the value, the more satisfied the respondent teacher is with his/her current job as a teacher. Burnout was measured using the question “Are you tired of the teaching profession?” Responses were rated on a 4-point scale, where 1 means that the interviewed teachers never feel burned out and 4 means that they often feel burned out in the teaching profession. The higher the value, the stronger the sense of burnout.

#### 3.2.5 Moderator variable

The moderator variable was interpersonal relationships (relationship), and this variable was measured by the question: “Do you have close colleagues at this school?” Here, 1 means that the interviewed teacher has almost no close coworkers at school, and 2 means that the interviewed teacher has many close coworkers. The higher the value, the better the interpersonal relationships of the interviewed teachers at work.

### 3.3 Econometric modeling

#### 3.3.1 Benchmark regression

This paper sought to analyze in depth the impact of administrative pressure on teachers’ professional identity, excluding other social environment and demographic characteristics. The parameters to be estimated were therefore identified using the Ordered Probit Model (oprobit), with teachers’ professional identity as the dependent variable and administrative pressure as the independent variable (Equations 1,2). The Ordered Probit Model is a regression analysis method designed for ordinal categorical dependent variables, particularly suited for scenarios where the outcome is hierarchical or ranked (e.g., Likert-scale ratings). Rooted in latent variable theory, the model posits that observed ordinal outcomes (such as “low,” “medium,” or “high” levels of teachers’ professional identity) are determined by an unobserved continuous latent variable y . Coefficients reflect the marginal impact of predictors on the latent scale, while marginal effects quantify changes in the probability of belonging to each ordinal category due to variations in predictors. Compared to the Ordered Logit model, the Ordered Probit assumes a normally distributed error term, making it more appropriate for data where the latent propensity exhibits a symmetric bell-shaped distribution across categories. This model is widely adopted in policy evaluation and social science research to analyze nonlinear relationships while providing interpretable probabilistic outcomes.


(1) $$\text{identit}y^{*}=\beta_{0}+\beta_{1}\times {\text{adpress}}_{i}+x_{i}\theta +u_{i}+\varepsilon_{i}$$

(2) $$\text{identity}=\{\begin{array}{c} 1,\text{identit}y_{i}^{*}\leq k_{0}\\ 2,k_{0}<\text{identit}y^{*}\leq \\ 3,\text{identit}y^{*}\leq k_{2} \end{array}k_{1}$$

where $\text{identit}y_{i}^{*}$ is the latent variable of teachers’ professional identity, and $k_{0}$ , $k_{1}$ , $k_{2}$ are the intercept points that satisfy $k_{0}<k_{1}<k_{2}$ ; ${\text{adpress}}_{i}$ is administrative pressure, and $x_{i}$ is a significant control variable affecting teachers’ professional identity, including their personal characteristics, job characteristics, and school characteristics. Finally, $u_{i}$ is a fixed effect for the region and $\varepsilon_{i}$ is a random error term.

Because the estimated coefficient in the nonlinear model is not the marginal utility of the parameter, the marginal utility of each parameter still needed to be calculated to better measure the impact of the independent variable on the dependent variable. Considering the large number of values for the dependent variable ${\text{identity}}_{i}$ , the marginal utility corresponding to the dependent variable teachers’ professional identity at ${\text{identity}}_{i}$ =3 is reported to facilitate the interpretation and comparison of the regression results; ${\text{identity}}_{i}$ =3 means that teachers have a strong professional identity and are very willing to continue in the teaching profession. If $\partial p({\text{identify}}_{i}=3)|\partial {\text{adpress}}_{i}\leq 0$ , it means that administrative pressure significantly reduces teachers’ professional identity.

#### 3.3.2 Mechanism testing

To test empirically whether administrative pressure has an impact on teachers’ professional identity through job satisfaction and burnout, the mechanisms for administrative pressure’ s effect on teachers’ professional identity were examined by adopting the mediation effect model based on the baseline model (Equations 3,4):


(3) $$M_{i}=\beta_{2}+\beta_{3}\times \mathrm{adp}r{\mathrm{ess}}_{i}+x_{i}\theta +u_{i}+\varepsilon_{i}$$

(4) $$\text{identit}y^{*}=\beta_{4}+\beta_{5}\times {\text{adpress}}_{i}+\beta_{6}M_{i}+x_{i}\theta +u_{i}+\varepsilon_{i}$$

Where $M_{i}$ is the mediating variable, which are job satisfaction (*satisfaction*) and burnout (*burnout*), respectively.

#### 3.3.3 Test of moderating effects

To test empirically whether administrative pressure has an impact on teachers’ professional identity through the moderating effect of interpersonal relationships, an interaction term between the independent variable and the moderating variable was added to the baseline model ( ${\text{adpress}}_{i}\times T_{i}$ ). The presence of moderation was verified by the significance of the interaction term, and the moderating effect model was constructed as shown below.


(5) $$\begin{array}{l} \text{identit}y^{*}=\beta_{7}+\beta_{8}\times {\text{adpress}}_{i}+\beta_{9}\times T_{i}+\beta_{10}\\ \times \mathrm{adp}r{\mathrm{ess}}_{i}\times T_{i}+x_{i}\theta +u_{i}+\varepsilon_{i} \end{array}$$

Where $T_{i}$ is the moderator variable and represents the interpersonal relationship (*relationship*).

## 4 Empirical analysis

### 4.1 Descriptive statistics

Table 1 shows the descriptive statistics of the main variables. The mean value for professional identity (*identity*) is 2.118, which indicates that the interviewed teachers would not necessarily return to teaching if they had to choose a new career. The mean value for the independent variable administrative pressure (*adpress*) is 3.415, which indicates that, on average, the teachers in the sample are experiencing between average and relatively high levels of administrative pressure. Among the control variables, the average age of the teachers was 38 years old, the average teaching experience was around 16 years, and most teachers had at least a bachelor’ s degree. Regarding the external environment, most schools where the interviewed teachers worked had circular playgrounds and student cafeterias, which coincided with the educational development of the country at that time, thus ensuring a representative sample.

**Table 1:** Descriptive statistics for variables.

Variable	Variable name	Mean	Std. Dev.	Min	Max
*identity*	Teachers’ professional identity	2.118	0.625	1.0	3.0
*adpress*	Administrative pressure	3.415	0.907	1.0	5.0
*age*	Age	38.253	7.580	23.0	61.0
*tschool*	Time teaching at the school	10.684	6.962	1.0	31.0
*lteaching*	Teaching experience	16.105	8.487	1.0	40.0
*married*	Marital status	1.921	0.366	1.0	4.0
*organization*	How long on teaching staff	1.127	0.333	1.0	2.0
*education*	Highest level of education	5.367	0.718	4.0	7.0
*salary*	Attitudes towards school pay	2.661	1.020	1.0	5.0
*award*	Awards received	0.107	0.309	0.0	1.0
*hours*	Working hours	47.655	17.985	9.0	124.0
*task*	Teaching tasks	2.181	1.168	1.0	10.0
*achievement*	Classroom achievement	3.353	1.017	1.0	5.0
*scale*	The size of the school	1128.175	792.947	122.0	3470.0
*canteen*	Whether the school has a playground	2.199	0.835	1.0	3.0
*playground*	Whether the school has a canteen	1.134	0.340	1.0	2.0
*climate*	The learning climate at the school	3.603	0.849	1.0	5.0

### 4.2 Benchmark regression

An oprobit model was used to analyze the intrinsic link between administrative pressure and teachers’ professional identity. Table 2 shows the results of the benchmark regression. As shown in Table 2, columns (1), (2), and (3) include the regression results of the control variables for individual characteristics, job characteristics, and school characteristics, respectively. The independent variable administrative pressure (*adpress*) is significantly negative at the 1% level after controlling for individual, job, and school characteristics variables separately. Column (4) shows the regression results after adding these three levels of control variables simultaneously; administrative pressure is still significantly negative at the 5% level. This suggests that the greater the administrative pressure, the lower the teachers’ professional identity and the less likely they are to remain in the teaching profession [ $\beta_{1}=-0.128,p<0.05$ , Table 2, column (4)]. Column (5) shows the marginal utility of teachers’ professional identity ( identity =3). When all covariates are at the mean, a one-unit increase in *adpress* decreases the probability that an interviewed teacher would definitely choose to remain in the teaching profession by 0.034. This again proves that administrative pressure has a significant negative effect on teachers’ professional identity, and that too much administrative pressure significantly reduces the likelihood that the interviewed teachers would remain in the teaching profession. The regression results validate H1.

**Table 2:** Effect of administrative pressure on teachers’ professional identity.

Variable	(1) *identity*	(2) *identity*	(3) *identity*	(4) *identity*	(5) *identity* = 3
*adpress*	−0.152^***^ (0.052)	−0.224^***^ (0.051)	−0.230^***^ (0.049)	−0.128^**^ (0.058)	−0.034^**^ (0.015)
*age*	−0.018 (0.016)			−0.014 (0.017)	−0.004 (0.004)
*tschool*	−0.016^*^ (0.009)			−0.019^*^ (0.010)	−0.005^*^ (0.003)
*lteaching*	0.001 (0.015)			0.001 (0.016)	0.000 (0.004)
*married*	0.135 (0.138)			0.230^*^ (0.135)	0.061^*^ (0.036)
*organization*	0.111 (0.163)			0.196 (0.173)	0.052 (0.045)
*education*	−0.045 (0.079)			−0.021 (0.089)	−0.006 (0.023)
*salary*	0.467^***^ (0.060)			0.402^***^ (0.067)	0.106^***^ (0.017)
*award*	−0.135 (0.158)			−0.010 (0.167)	−0.003 (0.044)
*hours*		0.003 (0.003)		−0.000 (0.003)	−0.000 (0.001)
*task*		0.035 (0.034)		0.001 (0.038)	0.000 (0.010)
*achievement*		−0.020 (0.043)		−0.051 (0.046)	−0.013 (0.012)
*scale*			−0.000 (0.000)	−0.000^**^ (0.000)	−0.000^**^ (0.000)
*canteen*			−0.225^***^ (0.073)	−0.226^***^ (0.087)	−0.060^**^ (0.023)
*playground*			−0.310 (0.191)	−0.365 (0.222)	−0.096^*^ (0.058)
*climate*			0.355^***^ (0.063)	0.333^***^ (0.075)	0.088^***^ (0.020)
*fixed effect*	YES	YES	YES	YES	YES
*N*	736	724	723	629	629

Values in parentheses are cluster-robust standard errors of regression coefficient estimates. * *p* < 0.10, ** *p* < 0.05, *** *p* < 0.01. Same as below.

Administrative pressure often manifests in the form of excessive workload, irrational administrative decisions, and imperfect management systems, which can increase teachers’ work pressure and uncertainty, and thus lead to a decline in their professional identity. Administrative pressure may also bring about rigidity in the education system, which can restrict teachers in terms of teaching content and methods, while preventing them from giving free play to their creativity. It may also lead teachers to believe that their work is not adequately supported and recognized by limiting their professional development and ultimately weakening their professional identity. Looking at the other control variables in the model revealed that when all else is equal, the longer the time spent teaching at the school and the larger the school size, the lower the teachers’ professional identity. When the school has a student cafeteria, however, the more satisfied the interviewed teachers are with the school system’s compensation package; also notable is that a better school learning culture can significantly increase teachers’ professional identity.

### 4.3 Heterogeneity analysis

To further explore the differences in the effects of administrative pressure on teachers’ professional identity, the samples were grouped according to teachers’ gender and the region in which the school was located. The effect of administrative pressure on teachers’ professional identity between different genders and different regions was tested using the oprobit model. The full sample was divided into male and female teachers by gender; the regression results are presented in Table 3. Columns (1) and (3) are the regression coefficients for male and female teachers, respectively; Columns (2) and (4) show the marginal effects for male and female teachers, respectively. As outlined in Table 3, the regression coefficient for administrative pressure is insignificant in the sample of male teachers and significantly negative at the 10% level in the sample of female teachers [ $\beta_{1}=-0.142,p<0.1$ , Table 3, column (3)]. This indicates that administrative pressure significantly reduces female teachers’ professional identity, while the effect of administrative pressure on male teachers is insignificant [ $\beta_{1}=-0.052,p>0.1$ , Table 3, column (1)].

**Table 3:** Split-sample regression estimate and marginal utility by gender.

Variable	Male		Female	
	(1)	(2)	(3)	(4)
	*identity*	*identity* = 3	*identity*	*identity* = 3
*adpress*	−0.052 (0.111)	−0.010 (0.022)	−0.142^*^ (0.074)	−0.039^*^ (0.020)
*control variable*	YES	YES	YES	YES
*fixed effect*	YES	YES	YES	YES
*N*	195	195	432	432

This phenomenon may be related to social gender role stereotypes and social expectations. Women are traditionally structured in society to take on family and childcare responsibilities, which can lead to multiple pressures on them from society, the family, and the workplace. Female teachers may also feel unfairly treated at work due to gender stereotypes inherent in society. This unfair treatment prevents them from feeling fully recognized and respected, thus further reducing their professional identity. In contrast, men are often seen as the family breadwinner and symbol of career success, and male teachers are more likely to receive support and recognition in the workplace. It is not surprising, then, with higher administrative pressure, female teachers are more likely to lose their professional identity than male teachers.

Table 4 shows the results of the regression estimation according to the region in which the school belongs. Schools were categorized into rural and non-rural areas. As detailed in the Table 4, the regression results show that both the regression coefficient and the marginal utility of administrative pressure in rural areas are significantly negative at the 1% level [ $\beta_{1}=-0.405,p<0.01$ , Table 4, column (1)], while neither the regression coefficient nor the marginal utility of administrative pressure in non-rural areas was significant [ $\beta_{1}=-0.077,p>0.1$ , Table 4, column (3)]. This suggests that administrative pressures in rural areas can significantly reduce teachers’ professional identity. Educational resources in rural areas are relatively scarce, the teaching and living environments of teachers are difficult, and their remuneration is relatively low. Under such circumstances, it is not surprising that rural teachers’ professional identity would be seriously weakened once administrative pressure in the school becomes too intense.

**Table 4:** Split-sample regression estimate and marginal utility by region.

Variable	Rural areas		Non-rural areas	
	(1)	(2)	(3)	(4)
	*identity*	*identity* = 3	*identity*	*identity* = 3
*adpress*	−0.405^***^ (0.140)	−0.081^***^ (0.025)	−0.077 (0.066)	−0.020 (0.017)
*control variable*	YES	YES	YES	YES
*fixed effect*	YES	YES	YES	YES
*N*	143	143	486	486

### 4.4 Discussion of endogeneity

The econometric model used here may suffer from endogeneity problems, which can lead to biased estimates of the regression coefficients as well as the marginal utility of administrative pressure on teachers’ professional identity. Endogeneity could stem from two main sources. First, there may be a bidirectional causal relationship between administrative pressure and teachers’ professional identity. Teachers with a stronger sense of professional identity have greater confidence in their willingness to remain in the teaching profession, which may result in far lower perception of administrative pressure than that felt by other teachers. Second, although the paper controlled for as many variables as possible, it is still possible that some variables may have been omitted, which would lead to the problem of self-selection bias if unobservable factors that affect both administrative pressure and teachers’ professional identity are not controlled for. To mitigate the interference of bidirectional causality and omitted variables on the estimation results, the conditional mixed process (CMP) method was adopted to further address the endogeneity problem and accurately obtain the causal effect of administrative pressure on teachers’ professional identity. The CMP method compares favorably to the traditional two-stage least squares method, which works best when both the independent and endogenous variables are estimated as ordered variables (Roodman, 2011). When using the CMP method for parameter estimation of endogenous variables, it is also necessary to find reasonable instrumental variables. The mean value of administrative pressure of other teachers in the same area was therefore used as an instrumental variable for the interviewed teachers.

The administrative pressure experienced by the interviewed teachers was closely related to that experienced by other teachers in the district because of the consistency of administrative decisions and educational management systems in the same region and the convergence of administrative pressure perceived by teachers in the same region. This instrumental variable is therefore correlated with the endogenous independent variables. The administrative pressure on other teachers in the same district does not, however, directly affect the professional identity of the interviewed teachers, so the instrumental variable is relatively exogenous.

Table 5 reports the results of the effect of administrative pressure on teachers’ professional identity obtained using the CMP method. Column (1) is the result of the Phase I regression; the dependent variable is administrative pressure of the interviewed teachers. Column (2) shows the results of the Phase II regression, where the dependent variable is the respondent teachers’ professional identity. Column (3) shows the marginal utility of administrative pressure on teachers’ professional identity. In this case, the result of the Kleibergen-Paap rk LM test is 186.808, which significantly rejects the original hypothesis. This indicates that the selected instrumental variables are correlated with the endogenous independent variables. The Cragg-Donald Wald F-statistic of 9956.437 is significantly greater than the Stock-Yogo weak instrumental variable test 10% threshold, which indicates that the model does not have a weak instrumental variable problem. As shown in Table 5, the results of the first stage of the regression show that administrative pressure is positively correlated with the administrative pressure on teachers in the same district [Table 5, column (1)]. In the second stage, administrative pressure negatively affects teachers’ professional identity, and the coefficient of the marginal utility remains significantly negative [Table 5, column (2)–(3)]. Overall, administrative pressure based on the CMP method still significantly reduces teachers’ professional identity after accounting for endogeneity.

**Table 5:** Conditional mixed process estimates of the effect of administrative pressure on teachers’ professional identity.

Variable	Phase I	Phase II	Marginal utility
	(1)	(2)	(3)
	*adpress*	*identity*	*identity* = 3
*adpress*		−0.596^**^ (0.279)	−0.095^***^ (0.034)
*adpress_iv*	0.489^***^ (0.168)		
*control variable*	YES	YES	YES
*fixed effect*	YES	YES	YES
*N*	789	789	789

### 4.5 Robustness test

To test the robustness of the effect of administrative pressure on teachers’ professional identity, the causal differences of administrative pressure on teachers’ professional identity are further assessed by replacing the dependent variable, the independent variable, and the estimated regression model. The regression results are shown in Table 6, where Column (1) replaces the dependent variable with “If you were to choose again, would you still choose to be a middle school teacher?” [ $\beta_{1}=-0.141,p<0.05$ , Table 6, column (1)]. Column (2) replaces the independent variable with “Are you satisfied with the way your school is managed?” Typically, the less administrative pressure teachers experience, the more satisfied they are with the way their school is managed [ $\beta_{1}=0.172,p<0.05$ , Table 6, column (2)]. Column (3) presents the regression results after re-estimation using an ordered logit model [ $\beta_{1}=-0.228,p<0.05$ , Table 6, column (3)]. The results of all three robustness tests strongly suggest that the inhibitory effect of administrative pressure on teachers’ professional identity is robust.

**Table 6:** Robustness test.

Variable	(1)		(2)		(3)	
	Replace dependent variable		Replace independent variable		Replace modeling approach	
	*midentity*	*midentity* = 3	*identity*	*identity* = 3	*identity*	*identity* = 3
*adpress*	−0.141^**^ (0.058)	−0.032^**^ (0.013)			−0.228^**^ (0.104)	−0.034^**^ (0.016)
*manage*			0.172^**^ (0.077)	0.045^**^ (0.020)		
*control variable*	YES	YES	YES	YES	YES	YES
*fixed effect*	YES	YES	YES	YES	YES	YES
*N*	630	630	628	628	629	629

## 5 Further analysis

### 5.1 Mediation analysis

In this study, how administrative pressure affects teachers’ professional identity was explored through mediation analysis. Based on the previous analysis, job satisfaction and burnout were selected as mediators to test the channels of influence of administrative pressure on teachers’ professional identity. Table 7 shows the results of the test of job satisfaction as a mediator. As detailed in Table 7, the results in Column (1) show that the regression coefficient of administrative pressure is significant at the 1% statistical level and the estimated coefficient is negative. This indicates that administrative pressure significantly reduces teachers’ job satisfaction [ $\beta_{3}=-0.311,p<0.01$ , Table 7, column (1)]; the regression results in Column (3) indicate that the mediation of job satisfaction is significant [ $\beta_{6}=0.491,p<0.01$ , Table 7, column (3)]. Job satisfaction has a full mediation effect, which indicates that administrative pressure can inhibit teachers’ professional identity by reducing their job satisfaction.

**Table 7:** Mechanism: job satisfaction.

Variable	Job satisfaction		Teachers’ professional identity	
	(1)	(2)	(3)	(4)
	*satisfaction*	*satisfaction* = 5	*identity*	*identity* = 3
*adpress*	−0.311^***^ (0.063)	−0.026^***^ (0.006)	−0.030 (0.065)	−0.007 (0.016)
*satisfaction*			0.491^***^ (0.073)	0.123^***^ (0.018)
*control variable*	YES	YES	YES	YES
*fixed effect*	YES	YES	YES	YES
*N*	630	630	628	628

Table 8 shows the results of the test of burnout as a mediator. As outlined in Table 8, the results in Column (1) show that the regression coefficient of administrative pressure is significantly positive at the 1% level, which indicates that there is a significant positive effect of administrative pressure on teachers’ burnout [ $\beta_{3}=0.254,p<0.01$ , Table 8, column (1)]. The regression results in Column (3) indicate that the mediation of teacher burnout is significant; burnout has a full mediation effect, which indicates that administrative pressure can inhibit teachers’ professional identity by increasing burnout [ $\beta_{6}=-0.886,p<0.01$ , Table 8, column (3)]. This analysis suggests that administrative pressure ultimately inhibits teachers’ professional identity by decreasing job satisfaction and increasing burnout. The regression results validate H2.

**Table 8:** Mechanism: burnout.

Variable	Burnout		Teachers’ professional identity	
	(1)	(2)	(3)	(4)
	*burnout*	*burnout* = 4	*identity*	*identity* = 3
*adpress*	0.254^***^ (0.051)	0.032^***^ (0.007)	−0.003 (0.061)	−0.001 (0.014)
*burnout*			−0.886^***^ (0.087)	−0.204^***^ (0.017)
*control variable*	YES	YES	YES	YES
*fixed effect*	YES	YES	YES	YES
*N*	631	631	629	629

### 5.2 Moderation analysis

According to H3, interpersonal relationships can have an important moderation effect on the relationship between administrative pressure and teachers’ professional identity. The cross-multiplier terms of interpersonal relationships and administrative pressure were generated according to Equation 5 and added to the regression equation to re-examine the moderation effect. The results of the moderation effects test are shown in Table 9; As shown in Table 9, column (1) shows the regression results for the effect of adding the cross-multiplier terms of administrative pressure and interpersonal variables on teachers’ professional identity. Column (2) shows the marginal utility. The regression coefficient of the cross-multiplier term is significantly positive at the 5% level, which indicates that the interpersonal relationship variable has a significant positive moderating effect [ $\beta_{10}=0.214,p<0.05$ , Table 9, column (1)]. When teachers have better interpersonal relationships, the inhibitory effect of administrative pressure on teachers’ professional identity declines. The regression results thus validated H3.

**Table 9:** Moderation effect: interpersonal relationships.

Variable	Teachers’ professional identity	
	(1)	(2)
	*identity*	*identity* = 3
*adpress*	−0.843^**^ (0.354)	−0.222^**^ (0.092)
*relationship*	−0.580 (0.390)	−0.153 (0.102)
*adpress × relationship*	0.214^**^ (0.105)	0.056^**^ (0.027)
*control variable*	YES	YES
*fixed effect*	YES	YES
*N*	628	628

## 6 Conclusions, discussion and implications

### 6.1 Conclusions and discussion

Enhancing teachers’ professional identity is of great significance for achieving high-quality development of education. Based on the 2014–2015 CEPS data, this paper empirically investigated the impact of administrative pressure on teachers’ professional identity and its mechanism of action. The results indicated, first, that administrative pressure has a negative effect on and significantly reduces teachers’ professional identity. After three sets of control variables—individual characteristics, job characteristics, and school characteristics—were added separately and simultaneously, this conclusion continued to hold.

Second, CMP was used to assess endogeneity, and three robustness tests were conducted: by replacing the dependent variables, by replacing the core explanatory variables, and by transforming the regression model. The results all indicate that administrative pressure significantly reduces teachers’ professional identity. Third, heterogeneity analyses showed that the inhibitory effect of administrative pressure on professional identity was more pronounced among female teachers, as well as among teachers from rural areas. Fourth, a mechanism test based on mediating effects revealed that administrative pressure can inhibit teachers’ professional identity through two channels: decreasing job satisfaction and increasing burnout. Fifth, tests based on the moderation effect indicated that interpersonal relationships have a positive moderation effect: When teachers have better interpersonal relationships, the inhibitory effect of administrative pressure on teachers’ professional identity is weaker.

This study investigates the impact of administrative pressure on teachers’ professional identity, emphasizing the positive moderating role of interpersonal relationships. This approach enables an examination of the factors influencing teachers’ professional identity from an administrative perspective, addressing existing research gaps related to administration, interpersonal relationships, and professional identity. Our findings reveal that administrative pressure negatively affects teachers’ professional identity. This can be attributed to the increase in non-teaching tasks resulting from such pressure, which consume a substantial portion of teachers’ time and energy. Consequently, teachers have fewer opportunities for teaching, research, and meaningful interactions with students-activities crucial for the development of their professional identity. When these fundamental responsibilities are compromised, teachers’ professional identity diminishes. Hypothesis 1 proposed that administrative pressure has a dampening effect on teachers’ professional identity. This hypothesis was strongly supported by the results [ $\beta_{1}=-0.128,p<0.05$ , Table 2, column (4)], indicating that higher perceived administrative pressure is associated with a diminished sense of teachers’ professional identity. Our findings are similar to Hendrikx’s (2019). Hendrikx (2019) found that institutional pressures, such as public management reform, could lead to substantial role changes for teachers, impacting their professional identity. Kelly and Barrett (2011) examined occupational stress experienced by accounting interns in Ireland and its effects on professional identity, concluding that occupational stress can significantly undermine professional identity. Furthermore, studies by Zhao et al. (2023) and Liang et al. (2022) corroborate that increased job stress among teachers is associated with a notable decline in both professional identity and occupational well-being. The development of professional identity among Pre-K teachers has been linked to administrative initiatives in early childhood care and education (Akaba et al., 2022). Our results underscore administrative pressure as a critical factor for teachers, particularly in contexts where bureaucratic responsibilities increasingly outweigh instructional priorities.

Our findings suggest that administrative pressure can negatively impact teachers’ professional identity through two primary mechanisms: decreased job satisfaction and increased burnout. The results strongly support hypothesis 2. As shown in Tables 7, 8, administrative pressure significantly reduces job satisfaction ( $\beta_{3}=-0.311,p<0.01$ ) and increases burnout ( $\beta_{3}=0.254,p<0.01$ ). Furthermore, job satisfaction and burnout exhibit a full mediation effect (job satisfaction: $\beta_{6}=0.491,p<0.01$ ; burnout: $\beta_{6}=-0.886,p<0.01$ ), which aligns with previous research. Previous research has established a significant association between teacher burnout and professional identity (Xing, 2022). In a survey study encompassing 9,058 Argentine teachers, Vargas Rubilar and Oros (2021) observed that elevated perceived stress during the COVID-19 pandemic correlated with higher rates of teacher burnout. The results underscore the complexity of pressure-identity relationships, urging educators to consider both emotional exhaustion and resource loss when designing interventions. Moreover, our study revealed that interpersonal relationships positively moderated the association between administrative pressure and teachers’ professional identity. Hypothesis 3 proposed that interpersonal relationships would moderate the negative effect of administrative pressure on teachers’ professional identity, indicating that the link between pressure and identity would be weaker among individuals with stronger relationships. This hypothesis was fully supported by the data (interaction term $\beta_{10}=0.214,p<0.05$ , Table 9). This moderating effect aligns with Social Support Theory, which posits that relational resources buffer the impact of stressors by providing emotional support, information, and instrumental aid, thereby reducing the perceived threat of administrative demands to teachers’ professional identity. It is well-established that social support contributes to positive psychological outcomes; when individuals receive interpersonal support from colleagues, family, and friends, it facilitates their attachment to the organization and enhances their professional identity (Lambert et al., 2016; Kim and Stoner, 2008). Furthermore, Schaap et al. (2021) corroborate that collegiality positively influences the professional identity of teachers transitioning in and out of secondary education careers in the Netherlands. The mechanism involved may encompass two pathways: first, positive interpersonal relationships cultivate a sense of belonging and mutual understanding, which can make administrative tasks feel less isolating; second, colleagues may exchange strategies for managing administrative burdens, thereby alleviating their emotional and cognitive toll. Notably, the significant interaction effect underscores that interpersonal dynamics are not merely correlated with identity but actively influence how stress is appraised and managed.

This study enhances the theoretical framework of professional identity, expands the research scope within educational psychology, and facilitates the integration of interdisciplinary theories, providing novel insights into the mechanisms underlying professional identity formation. From a practical standpoint, these findings can guide educational administrators in creating a more supportive environment for teachers’ professional growth by implementing strategies such as reducing administrative burdens and streamlining management processes. Moreover, our results emphasize the significance of fostering a positive interpersonal atmosphere within educational institutions and promoting communication and collaboration among educators to reinforce their sense of belonging and professional identity.

### 6.2 Implications and limitations

The results yield several suggestions to further enhance teachers’ professional identity and cultivate a high-quality teaching force. First, school administrations should standardize the boundaries for teachers to exercise their rights, improve the efficiency of administrative management, and reduce the administrative pressure on teachers. Currently, teachers not only have to undertake educational and teaching tasks, but they also have to handle many administrative tasks and other non-teaching work, which increases their workload and sometimes interferes with their teaching activities. Excessive administrative pressure and unequal treatment generally leave teachers professionally exhausted and reduce their professional identity. By clarifying rights and responsibilities, rationalizing the division of labor, and reducing redundant and ineffective work, the administrative pressure on teachers can be greatly reduced, so they are more likely to have more energy to devote to teaching, thus enhancing their professional identity.

Second, the education administration should introduce targeted measures to enhance the professional identity of female and rural teachers. Management should create a workplace environment that is fair and just and respects women, as well as fully recognizing the educational and teaching abilities of female teachers and providing fair opportunities for career development as well as vocational training and guidance that is suitable for women. The difficulties of female teachers in balancing their families and careers would be solved by establishing flexible working hours and providing benefits such as maternity and parental leave; this would also strengthen their professional identity.

The professional status and treatment of rural teachers should be improved through measures such as raising salaries, improving the working environment, and enhancing vocational and teacher training. The gap between urban and rural education should be reduced by strengthening the equitable distribution of educational resources, so that rural teachers have more opportunities for development. This would enhance their professional identity, while also ensuring a high-quality education for rural students.

Finally, schools should create an atmosphere of good interpersonal relationships among teachers. This could involve increasing opportunities for communication and cooperation among teachers through team teaching and project cooperation, as well as establishing effective collaborative relationships. Trainings in communication skills should be organized to create an open and accepting work environment and help teachers develop shared values and a sense of mission. Regular staff activities should be organized to stimulate teachers’ intrinsic motivation to interact. Regular mental health training could also be provided to help teachers channel their negative emotions, overcome psychological barriers, resolve interpersonal problems, and build a positive, sincere and harmonious attitude toward interpersonal interactions.

In conclusion, it is necessary to reduce administrative pressure on teachers and to shape good interpersonal relationships among them to improve their professional identity. The following is particularly true for female teachers and teachers in rural areas: It is important to treat them fairly and to respect their contribution to education. This may be the best way to fully enhance teachers’ professional identity and guarantee the quality of education.

This empirical study, like all such research, has certain limitations. Primarily, our data collection was constrained, particularly due to the challenges in obtaining cross-country data for continuous follow-up surveys. As a result, our focus was limited to the Chinese sample. However, it is important to note that the phenomenon of administrative pressure inhibiting teachers’ professional identity is not exclusive to China. It is observed in other countries, especially in emerging developing nations. This presents a promising avenue for future research to investigate the replicability of our findings in diverse national contexts. Furthermore, while our study examined the impact of administrative pressure on teachers’ professional identity, it could not identify all potential mechanisms of influence. Our analysis focused on two specific mechanisms: job satisfaction and burnout. We posit that a more comprehensive investigation of these issues could yield significant and relevant conclusions, thereby establishing a foundation for future research in this area.

## Data Availability

The original contributions presented in the study are included in the article/supplementary material, further inquiries can be directed to the corresponding author.
